# Symbolic Recurrence Analysis of RR Interval to Detect Atrial Fibrillation

**DOI:** 10.3390/jcm8111840

**Published:** 2019-11-02

**Authors:** Jesús Pérez-Valero, M. Victoria Caballero Pintado, Francisco Melgarejo, Antonio-Javier García-Sánchez, Joan Garcia-Haro, Francisco García Córdoba, José A. García Córdoba, Eduardo Pinar, Arcadio García Alberola, Mariano Matilla-García, Paul Curtin, Manish Arora, Manuel Ruiz Marín

**Affiliations:** 1 Departamento de Tecnologías de la Información y las Comunicaciones, Campus la Muralla, Universidad Politécnica de Cartagena, Edif. Antigones, 30202 Cartagena, Spain; jesus.perez@edu.upct.es (J.P.-V.); antoniojavier.garcia@upct.es (A.-J.G.-S.); joang.haro@upct.es (J.G.-H.); 2 Departamento de Métodos Cuantitativos para la Economía y la Empresa, Campus de Espinardo, Universidad de Murcia, 30001 Murcia, Spain; mvictori@um.es; 3 Departamento de Cardiología, Hospital Universitario Virgen de la Arrixaca, 30120 Murcia, Spain; francisco.melgarejo@goumh.umh.es (F.M.); epbhva@yahoo.es (E.P.); arcadi@secardiologia.es (A.G.A.); 4 Unidad de Cuidados Intensivos, Hospital Universitario Los Arcos del Mar Menor, 30739 San Javier-Murcia, Spain; pagacor@gmail.com; 5 Departamento de Métodos Cuantitativos, Ciencias Jurídicas y Lenguas Modernas, Universidad Politécnica de Cartagena, Calle Real 3, Edif. CIM, 30202 Cartagena, Spain; josea.garcia@upct.es; 6 Facultad de Ciencias Económicas y Empresariales, Universidad Nacional de Educación a Distancia, Paseo Senda del Rey, 11, 28040 Madrid, Spain; mmatilla@cee.uned.es; 7 Icahn School of Medicine, 11 Mount Sinai, One Gustave L Levy Place, Box 1057, New York, NY 10029, USA; paul.curtin@mssm.edu (P.C.); manish.arora@mssm.edu (M.A.)

**Keywords:** atrial fibrillation, symbolic analysis, symbolic recurrence quantification analysis, logistic model

## Abstract

Atrial fibrillation (AF) is a sustained cardiac arrhythmia associated with stroke, heart failure, and related health conditions. Though easily diagnosed upon presentation in a clinical setting, the transient and/or intermittent emergence of AF episodes present diagnostic and clinical monitoring challenges that would ideally be met with automated ambulatory monitoring and detection. Current approaches to address these needs, commonly available both in smartphone applications and dedicated technologies, combine electrocardiogram (ECG) sensors with predictive algorithms to detect AF. These methods typically require extensive preprocessing, preliminary signal analysis, and the integration of a wide and complex array of features for the detection of AF events, and are consequently vulnerable to over-fitting. In this paper, we introduce the application of symbolic recurrence quantification analysis (SRQA) for the study of ECG signals and detection of AF events, which requires minimal pre-processing and allows the construction of highly accurate predictive algorithms from relatively few features. In addition, this approach is robust against commonly-encountered signal processing challenges that are expected in ambulatory monitoring contexts, including noisy and non-stationary data. We demonstrate the application of this method to yield a highly accurate predictive algorithm, which at optimal threshold values is 97.9% sensitive, 97.6% specific, and 97.7% accurate in classifying AF signals. To confirm the robust generalizability of this approach, we further evaluated its performance in the implementation of a 10-fold cross-validation paradigm, yielding 97.4% accuracy. In sum, these findings emphasize the robust utility of SRQA for the analysis of ECG signals and detection of AF. To the best of our knowledge, the proposed model is the first to incorporate symbolic analysis for AF beat detection.

## 1. Introduction

Atrial fibrillation (AF) is the most common sustained arrhythmia associated with a high risk of an ischemic stroke [1]. For this reason, timely diagnosis of arrhythmia is of crucial importance. The diagnosis of AF can be difficult since patients may be asymptomatic, particularly in cases of intermittent AF episodes that end spontaneously (paroxysmal atrial fibrillation, PAF). Given the significant risk of mortality and morbidity, and the fact that asymptomatic AF is not detected unless specifically looked for, there is a strong impetus for ambulatory monitoring. With the greater need for ambulatory monitoring, accurate and automated detection of asymptomatic AF becomes a relevant task. AF is usually diagnosed by inspection of the electrocardiogram (ECG) where it becomes apparent in the dysregulation of ECG signal properties. AF is characterized in the ECG by loss of the normal atrial depolarization waves (P waves), rapid and irregular atrial fibrillatory waves with an undulating baseline and typically irregular RR intervals (time between two consecutive ventricular beats) [2]. 

In this paper, we propose to use a symbolic recurrence approach to detect AF. Symbolic Recurrence Quantification Analysis (SRQA) [3] offers a powerful framework to investigate complexity in dynamical systems from their time series, without knowledge of their underlying governing equations. It is especially useful when the dynamical system is non-stationary, as may be commonly encountered in ECG signals, and is additionally advantageous in requiring the generation of a minimal descriptive feature set that allows the implementation of robust and simple predictive algorithms. Specifically, we construct a logistic regression algorithm that employs SRQA over RR interval time series to detect AF. Moreover, to show the robustness of this approach we perform a K-fold cross-validation procedure that provides excellent results in terms of model coefficients, sensitivity, specificity and accuracy. To the best of our knowledge, the proposed model is the first to incorporate symbolic analysis for AF beat detection. 

The rest of the paper is organized as follows. In Section 2, we introduce the basic definitions and notation for the development of symbolic recurrence measures and propose a logistic model for AF classification. In Section 3, we estimate the logistic model and study the classification power of the model in terms of sensitivity, specificity, and accuracy. Moreover, we report the results of a K-fold cross validation procedure for the estimated model. These results are summarized and discussed in Section 4. Finally, Section 5 concludes.

## 2. Materials and Methods

### 2.1. Symbolization

In general, given a real-valued time-series ${\left\{x_{t}\right\}}_{t=1}^{T}$, the phase space vectors can be reconstructed through ${\bar{x}}_{t}=\left(x_{t},x_{t+1},\ldots ,x_{t+m-1}\right)$ for an embedding dimension m. Let us denote by $S_{m}$ the symmetric group of order m!, that is the group formed by all the permutations of length m. Let $\pi =\left(i_{1},i_{2},\ldots ,i_{m}\right)\in S_{m}$. We will call an element π in the symmetric group $S_{m}$ a symbol. Notice that ${\left\{{\bar{x}}_{t}\right\}}_{t=1}^{n}$ is a vectorial time series of length n=T−m+1. Each ${\bar{x}}_{t}$ is called an m− history. 

We say that ${\bar{x}}_{t}$ is of $\pi_{i}-$ type if and only if $\pi_{i}=\left(i_{1},i_{2},\ldots ,i_{m}\right)$ is the unique symbol (permutation) in the group $S_{m}$ satisfying the two following conditions:

$$\begin{array}{c} \left(a\right)\;x_{t+i_{1}}\leq x_{t+i_{2}}\leq \cdots \leq x_{t+i_{m}},\;\mathrm{and}\\ \left(b\right)\;i_{s-1}<i_{s}\;\mathrm{if}\;x_{t+i_{s-1}}=x_{t+i_{s}} \end{array}$$

Condition (b) guarantees uniqueness of the symbol π. Therefore, we have defined the symbolization map

$$S:{\mathbb{R}}^{m}⟶S_{m}$$

given by $S\left({\bar{x}}_{t}\right)=\pi$ if and only if ${\bar{x}}_{t}$ is of π− type. Note that the symbolization map S transforms the vectorial time series of m-histories in a sequence of symbols that correspond to the ordinal patterns of each consecutive m values of the time series. Moreover, each element of ${\mathbb{R}}^{m}$ is mapped to a symbol of $S_{m}$ providing a partition of ${\mathbb{R}}^{m}$ of size m!, called the symbolic partition of ${\mathbb{R}}^{m}$. The greater the parameter m is the more capacity of the symbolization map to gather more complex dynamic structure of the RR interval time series; but, on the other hand, the set of symbols grows dramatically with m. For m=3 we have 6 symbols; for m=4 there are 24 symbols; for m=5 the number of symbols is 120; and so on. For the symbolization procedure to be efficient the set of symbols should satisfy the following two conditions: (i) the number of symbols has to be smaller than the length of the time series to be symbolized and (ii) expected frequencies of symbols under independence should be ≥5 in order for the symbols to be statistically distinguishable with the $\chi^{2}$ distribution. Thus, in general the size of the window of RR interval data to be symbolized, namely w, has to satisfy that w≤5m!, which for windows of length w=30 admits a maximum value of m=3. Since the purpose of this paper is to detect AF on small windows of RR interval data (w=30,60,120, and 200 data points) we will fix m=3, that is 6 symbols. 

**Figure 1:**
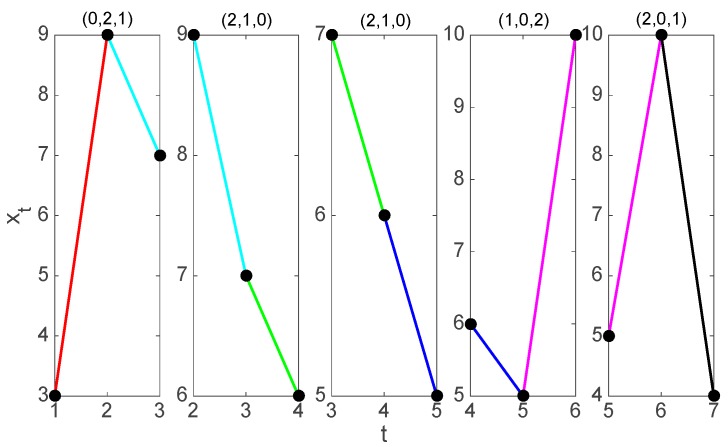
Symbolization of the time series of seven values given by (1) for m=3. From left to right we find the five 3-histories that can be constructed and their associated symbol.

We illustrate the symbolization procedure on the time series shown in Figure 1, whose seven values are

(1) $$\left\{x_{1}=3,x_{2}=9,x_{3}=7,x_{4}=6,x_{5}=5,x_{6}=10,x_{7}=4\right\}$$

For an embedding dimension m=3, we have six symbols forming the symmetric group, that is,

$$S_{3}=\left\{\left(0,1,2\right),\left(0,2,1\right),\left(1,0,2\right),\left(1,2,0\right),\left(2,0,1\right),\left(2,1,0\right)\right\}$$

Each one of the 3-histories generated from the time series given by (1) can be uniquely mapped into a symbol in $S_{3}$. For instance, for t=4, ${\bar{x}}_{4}=\left(6,\;5,\;10\right)$ we have that $x_{t+1}=5<x_{t+0}=6<x_{t+2}=10$, which implies that ${\bar{x}}_{4}$ is of (1,0,2)-type.

### 2.2. Symbolic Recurrence Analysis

We will say that two states ${\bar{x}}_{t}$ and ${\bar{x}}_{s}$ are symbolic recurrent states if and only if $S\left({\bar{x}}_{t}\right)=S\left({\bar{x}}_{s}\right)$. Therefore, we can define the following indicator function

(2) $${\mathrm{SR}}_{\mathrm{ts}}=\{\begin{array}{cc} 1 & \mathrm{if}\;S\left({\bar{x}}_{t}\right)=S\left({\bar{x}}_{s}\right)\\ 0 & \mathrm{otherwise} \end{array}$$

that always takes the value 1 when the ordinal patterns of the m-histories ${\bar{x}}_{t}$ and ${\bar{x}}_{s}$ are of the same type (symbolic recurrent). To distinguish recurrences among different symbols, for each symbol π we define

(3) $${\mathrm{SR}}_{\mathrm{ts}}\left(\pi \right)=\{\begin{array}{cc} 1 & \mathrm{if}\;S\left({\bar{x}}_{t}\right)=S\left({\bar{x}}_{s}\right)=\pi \\ 0 & \mathrm{otherwise} \end{array}$$

that takes the value 1 when the ordinal patterns of the m-histories ${\bar{x}}_{t}$ and ${\bar{x}}_{s}$ are both of π-type (symbolic recurrent to π). 

### 2.3. Symbolic Recurrence Plots of RR Interval Time Series

The indicator functions ${\mathrm{SR}}_{\mathrm{ts}}$ and ${\mathrm{SR}}_{\mathrm{ts}}\left(\pi \right)$ define symbolic recurrence n×n-matrices (SR and SR(π) respectively). Notice that $\mathrm{SR}=\sum_{\pi \in S_{m}}\mathrm{SR}\left(\pi \right)$. These matrices can be represented in a Symbolic Recurrence Plot (SRP), and Symbolic Recurrence Plot to a symbol π (SRP(π)) respectively. Symbolic recurrence plots illustrate when two m-histories belong to the same set of the symbolic partition and therefore have the same ordinal pattern. Thus, if a dynamic change occurs in the time series then the distribution of ordinal patterns will change, producing in turn a change in the symbolic recurrences of the m-histories and therefore, in the symbolic recurrence plot (see [3] for examples). 

**Figure 2:**
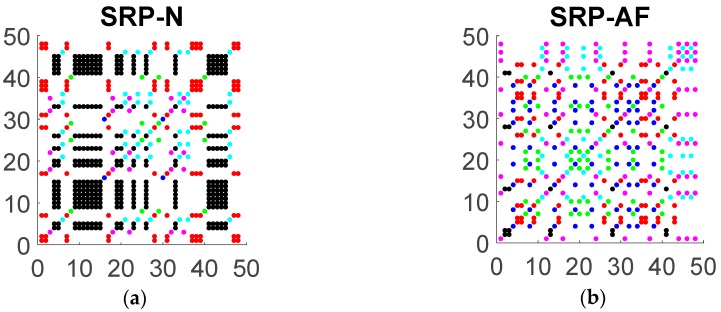
Symbolic recurrence plot (SRP) of heart beat interval RR for embedding dimension m=3 in normal sinus (**a**) and atrial fibrillation (**b**). N denotes normal sinus and AF atrial fibrillation.

Recall that in a symbolic recurrence plot (SRP) each colored dot represents recurrence to its corresponding symbol, with the coordinate axes reflecting the temporal interval between recurrences. Figure 2 illustrates two SRPs corresponding to a 50 RR interval data with embedding dimension m=3 (that is m!=6 symbols) of a patient classified as normal sinus (N) and a patient with atrial fibrillation (AF). The utility of the SRP is in visualizing both the preponderance of a given symbol type, reflecting the temporal distribution of that dynamic, but also in revealing the temporal organization of these dynamics. These structural dynamics, particularly the organization of diagonal lines, reflecting cyclical dynamics, and vertical/horizontal structures, indicative of periods of persistent symbolic dynamics, provide the basis for subsequent quantitative analyses to characterize the prevalence, duration, and complexity of symbolic dynamics in the ECG signal. 

**Figure 3:**
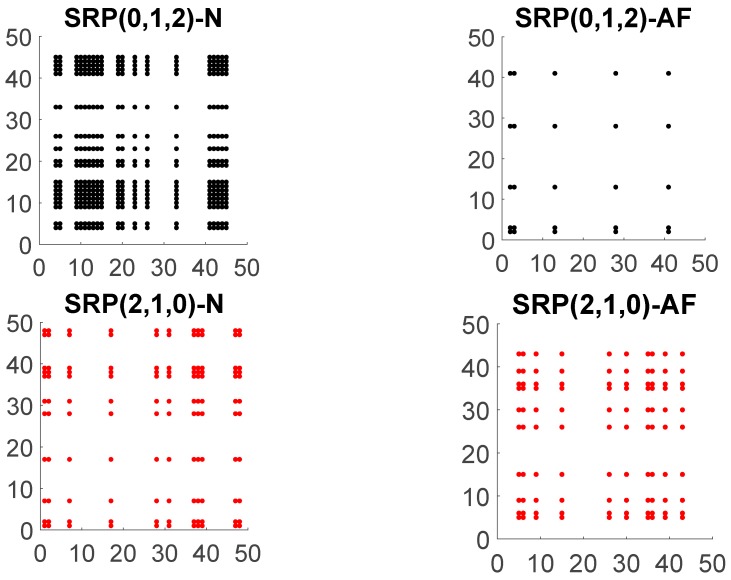
Symbolic recurrence plot (SRP) to increasing (first row) and decreasing (second row) symbols of an RR interval time series of length 50 for embedding dimension m=3 of a patient in normal sinus (**left**) and another patient in atrial fibrillation (**right**). N denotes normal sinus and AF atrial fibrillation.

The relevance of symbol distribution and organization in the SRP is apparent in comparing the SRP of the AF signal and N signal, as shown in Figure 3. Note, in particular, that the distribution of the colored points that conforms each SRP is different among normal sinus and atrial fibrillation patients which suggests a different complex dynamic behavior of the signal for each type of patient. 

The dominant color in the patient with normal sinus is black corresponding to the increasing symbol (0,1,2) followed by the red color that corresponds to the decreasing symbol (2,1,0). Moreover, the recurrences to the increasing symbol reveal a strong pattern shaping rectangles of larger area than the area of the rectangles formed by any other symbol. This does not happen for patients with AF. For the case of the patient with AF, we do not observe any regular pattern in the plot and the symbols seem to be randomly distributed. In Figure 3, we show the symbolic recurrence plots to the increasing and decreasing symbols for N and AF patients. 

### 2.4. Symbolic Recurrence Measures

Based on the previous symbolic recurrence plots, we define the following symbolic recurrence measures, which quantify both the distribution (type and number of recurrence symbols) and organization (structural properties, e.g., diagonal or vertical lines) of the SRP. The global symbolic recurrence rate is defined as

(4) $$\mathrm{SRR}=\frac{1}{n^{2}}\sum_{t,s=1}^{n}{\mathrm{SR}}_{\mathrm{ts}},$$

and the symbolic recurrence rate to each symbol $\pi \in S_{m}$ as

(5) $$\mathrm{SRR}\left(\pi \right)=\frac{1}{n^{2}}\sum_{t,s=1}^{n}{\mathrm{SR}}_{\mathrm{ts}}\left(\pi \right).$$

While the global SRR captures the rate at which symbolic dynamics repeats within a given signal, the symbol-specific SRR(π) focuses this metric specifically to a given symbol type. The remaining symbolic recurrence measures used as covariates in the logistic model are based on two types of structures in the SRP, namely, diagonal and vertical (or equivalently horizontal, for symmetry) lines. Specifically, we define the distributions {(d,n(d))} and {(v,n(v))} of diagonal and vertical lines of length at least 2, where n(d) and n(v) are the number of diagonal and vertical lines of length d and v, respectively. It should be noted that vertical lines only appear with the increasing and decreasing symbols, since the remaining symbols can not appear two consecutive times. Also, when examining the diagonal lines, we neglect the main diagonal that is always composed of all ones. 

Structures in SRPs provide useful evidence about the dynamic behavior of the system. Diagonal lines of length d between (t,s) and (t+d,s+d) identify a sequence of symbols whose phase space vectors $\left({\bar{x}}_{t},{\bar{x}}_{s}\right)$, $\left({\bar{x}}_{t+1},{\bar{x}}_{s+1}\right)$, …, $\left({\bar{x}}_{t+d},{\bar{x}}_{s+d}\right)$ belong to the same set in the partition of the phase space, consistent with a periodic trajectory. Such a set could potentially vary along the sequence, whereby the two phase vectors could visit multiple subsets of the partition. In other words, diagonal lines represent two equal sequences of symbols of length d, one starting at time t and one at time s. Therefore, diagonal lines reveal some form of predictability of the system, with long diagonals indicating long repeated sequences of symbols. From the distribution of the diagonal lines, we can compute the percentage of recurrence points which form diagonal lines as

(6) $$D=\frac{\sum_{i=d_{\min}}^{d_{\max}}d_{i}n\left(d_{i}\right)}{n^{2}\mathrm{SRR}},$$

where, once again, we have neglected the main diagonal. This indicator is equivalent to the so-called determinism, scaled by the SRR.

Another important type of structure observed in SRPs is vertical lines, which correspond to a sequence of phase space vectors ${\bar{x}}_{t}$, ${\bar{x}}_{t+1}$, …, ${\bar{x}}_{t+v}$ that have the same ordinal pattern. Similar to classical recurrence plots, the length of the vertical lines quantifies the number of time instants in the time-series where the phase space vectors are constrained in the same set of the symbolic partition. We define the trapping time as the average vertical length $\bar{v}$. 

All these symbolic recurrence measures together with the entropy of the distributions of vertical and diagonal lines, can be used to analyze and characterize the dynamic behavior of the time series given by the RR interval according to the heart health state of the individual. For the case of the example depicted in Figure 2 and Figure 3, Table A1 (see Appendix A) specifies symbolic recurrence measures. These measures might become an efficient and powerful tool to discriminate between AF and N patients.

### 2.5. A logistic Model to Clasify AF Patients

In this section we propose a logistic model to estimate the probability of a patient to be classified as AF. Moreover, based on receiver operating characteristic curve analysis, a probability threshold is given so that a patient with an estimated probability above the threshold is classified as AF. 

The covariates used for the estimation of the logistic model are derived from SRQA analysis and the distribution of the RR interval and are divided into two groups. The first group is formed by symbolic recurrence measures SRR(π), D, ent(d), $\mathrm{rline}v_{\delta }$, $\mathrm{ent}\left(v_{\delta }\right)$, with δ=(0,1,2) or (2,1,0), $\pi \in S_{m}$ and m=3. The second group consist of two central tendency measures, the mean $\bar{\mathrm{RR}}$ and the median Me(RR), and two dispersion measures, Pearson coefficient of variation denoted by CV (standard deviation divided by the mean), and the coefficient of variation of the median which is computed as

(7) $$V_{\mathrm{Me}}=\frac{\sum_{i}|{\mathrm{RR}}_{i}-\mathrm{Me}\left(\mathrm{RR}\right)|}{\sum_{i}{\mathrm{RR}}_{i}}.$$

Both CV and $V_{\mathrm{Me}}$ are homogeneity measures that account for the variability of the RR interval time series. 

### 2.6. Data

Each RR interval time series is obtained from the ECG signals taken from PhysioNet MIT-BIH Atrial Fibrillation Database [4]. This data base consists of a total of 25 patients with 149.06 h NS, 93.77 h AF and 6.6 h of other arrhythmias (such as atrial flutter and AV junctional rhythm). Each record is divided into consecutive windows containing 30, 60, 120, or 200 RR intervals. Predictive models were built using covariates calculated for each window, and classification results were compared to annotations. A window was labelled true AF if more than 1/2 of cycles in the window were annotated as AF and non-AF otherwise.

## 3. Results

### 3.1. Model Estimation

We found that covariates $\bar{\mathrm{RR}}$, Me(RR), CV, $V_{\mathrm{Me}}$, SRR(π) for all $\pi \in S_{3}$, and ent(d) were always significant for all windows sizes, with a p-value <0.001. Covariates D and $\mathrm{ent}\left(v_{\left(0,1,2\right)}\right)$ were not significant for the smallest windows of size w=30, $\mathrm{ent}\left(v_{\left(2,1,0\right)}\right)$ is not significant for the smaller and greater windows sizes w=30,200 respectively, and finally ${\bar{v}}_{\left(0,1,2\right)}$ and ${\bar{v}}_{\left(2,1,0\right)}$ are not significant for any window size. These findings indicate that the most reliable discriminative factors in classifying AF signals are related to either global measures of central tendency (e.g., $\bar{\mathrm{RR}}$, Me(RR), CV, $V_{\mathrm{Me}}$), or to the distribution of specific symbol recurrence rates. Measures of structural organization, such as determinism and entropy, were also reliable indicators of AF, but the utility of these measures was dependent on a longer sampling window (w>30). Interestingly, the organization of vertical structures in the SRPs, indicative of periods of persistent symbolic dynamics, were not useful predictors of AF. These findings indicate that the separation of AF signals from NS signals is driven by diverging sequential dynamics and symbolic diversity rather than differences in persistent stable states. 

Table A2 (see Appendix A) specifies the coefficients of the logistic model for each window size (w). Coefficient with a superimposed ∗ and ∗∗ are significant at 5% and 1% respectively, while those without asterisk mark are considered non-significant. 

### 3.2. Classification Power of the Model

In order to show the classification power of the model we computed its specificity, sensitivity, and accuracy with respect to predicting AF status. To this end we define

- TP is the number of true positive classified by the model.
- TN is the number of true negative classified by the model.
- FN is the number of false negative classified by the model.
- FP is the number of false positive classified by the model.
- Se is the true positive rate computed as $\mathrm{Se}=\frac{\mathrm{TP}}{\mathrm{TP}+\mathrm{FN}}$, also known as sensitivity.
- FPR is the false positive rate computed as $\mathrm{FPR}=\frac{\mathrm{FP}}{\mathrm{TN}+\mathrm{FP}}$. Specificity is known as Sp=1−FPR which measures the proportion of actual negatives that are correctly identified by the model as such.
- $\mathrm{ACC}=\frac{\mathrm{VP}+\mathrm{VN}}{\mathrm{VP}+\mathrm{FN}+\mathrm{FP}+\mathrm{VN}}$ determines the model accuracy.

To calculate the threshold for model classification, namely τ, such that a patient with an estimated probability above τ is considered AF we rely on ROC (Receiver Operating Characteristics) curves. More concretely, for each threshold τ from 0 to 1 at steps of 0.001 we compute the points ${\left\{\left({\mathrm{FPR}}_{\tau },{\mathrm{Se}}_{\tau }\right)\right\}}_{\tau }$ that form the ROC curve. The optimal case is the one in which $\left({\mathrm{FPR}}_{\tau },{\mathrm{Se}}_{\tau }\right)=\left(0,1\right)$, that is, zero false positives and zero false negatives. Then, the threshold is taken as the one that minimizes the distance between the points $\left({\mathrm{FPR}}_{\tau },{\mathrm{Se}}_{\tau }\right)$ and (0,1),

$$\tau =\arg\min_{\tau }\left\{{\mathrm{FPR}}_{\tau }^{2}+{\left(1-{\mathrm{Se}}_{\tau }\right)}^{2}\right\}$$

The values of τ for each window size together with sensitivity, specificity, and accuracy are given in Table 1.

As expected, the sensitivity, specificity, and accuracy of the model increase with the size of the window. Sensitivity always remains above 96%, reaching 97.9% for windows of size 200. Regarding specificity, the power performance of the algorithm is slightly lower although still with very good performance with values from 94.8% for the smallest window size and reaching 97.6% for windows of size 200. Finally, the predictive power of the model (accuracy) is also very high, ranging from 95.4% to 97.7% depending on the window size. 

### 3.3. Model Validation

In order to show the robustness and consistency of symbolic recurrence measures as predictor of AF in a logistic model we have performed a K-fold cross-validation procedure. This procedure has only one parameter K and consists of splitting the data set into K subsets. Then for each unique subset we take the subset as a hold out or test data set and the remaining subsets as a training data set. We fit the logistic model on the training set and evaluate it on the test set. Afterward we retain the evaluation score (Se, Sp, and ACC) and discard the model. We have chosen K=10, a value that has been found through experimentation to generally result in a model skill estimate with low bias and modest variance. 

Table 2 shows the median (Me) and percentiles 25th and 75th (P_25_ and P_75_ respectively) values of the threshold, Sensitivity, Specificity, and Accuracy of the 10-fold cross-validation procedure for each window size. 

As shown in Table 2, the classification power of the logistic model does not depend on the robustness of the training data.

## 4. Discussion

Here, we characterized the use of symbolic recurrence quantification analysis (SRQA) [3] to generate a predictive algorithm for the detection of cardiac atrial fibrillation (AF). This approach requires minimal preprocessing, utilizing only the RR interval from the ECG waveform, and is robust against signal processing challenges that are likely to be common in an ambulatory signal detection setting, including noisy, non-stationary signals. We demonstrate the robust predictive performance of this model utilizing simple predictive algorithms that utilize relatively few features, and are therefore likely to generalize well and be minimally vulnerable to over-fitting, and validate this approach via a cross-validation paradigm.

There are many different algorithms to detect AF in the specialized literature, typically based on the analysis of ECG data. These include methods based on the analysis of P-waves via temporal or frequency domain methods, as in [5,6,7,8,9,10,11]; and, more recently, the application of machine learning and deep learning methods are increasingly popular due to their ability to automatically learn features at multiple levels of abstraction (i.e., layers). Andreotti et al., 2017 [12], for example, proposed the use of a convolutional neural network utilizing 169 features in a supervised learning strategy for AF detection. Similarly, a convolutional recurrent neural network was employed for AF detection by Liman and Precioso [13], while a decision tree ensemble was implemented by Bin et al. [14]. Though the predictive algorithms utilized in these approaches varied, a commonality in these approaches is in the use of a wide array of features for model training, and the use of 12-lead ECG records for signal acquisition during ambulatory detection. In contrast, the methodology introduced here focuses solely on the analysis of RR interval, which can be extracted with simpler sampling methods.

Alternative strategies have also been devised to detect AF based on just the RR interval. For example, Lian et al. [15] proposed a new AF detection moving window algorithm based on a map that plots RR intervals versus change of RR intervals. The map is divided by a grid with 25-ms resolution in two axes and nonempty cells are counted to classify AF and non-AF episodes. For each window size, and based on receiver operating characteristic curve analysis, a threshold is calculated for classification purposes, obtaining a high power performance of the algorithm. Similarly, in [16] authors used the coefficient of variation of standard density histograms of RR and ΔRR = RR_i_ − RR_i−1_ time series to detect atrial fibrillation. Further, density histograms of RR or ΔRR intervals in test data are compared with standard density histograms using the Kolmogorov-Smirnov test. If there is no significant difference between two given histograms, the rhythm is labeled as AF, obtaining high values of sensitivity and specificity. Another alternative strategy, introduced in [17], is based on the generally accepted characteristic of AF as a random sequence of heart beat intervals with markedly increased beat-to-beat variability and complexity. In order to exploit these characteristics, the authors developed an algorithm combining (i) the Root Mean Square of Successive RR Differences to quantify variability, (ii) the Turning Points Ratio to test for randomness of the time series, and (iii) Shannon entropy to characterize its complexity. Results of the method show high sensitivity and specificity. 

Nevertheless, the main drawbacks of these algorithms are that they depend on the robustness of the training data, require preliminary filtering in an initial preprocessing step, rely upon a complex array of features which may be computationally expensive to extract, and remain vulnerable to the estimation of false positive predictions. As well, the available literature where these procedures have been validated does not include extensive use of cross-validation procedures to show that the accuracy of AF detection is not compromised by the training set. These controls, however, are essential for the development of robust ambulatory monitoring algorithms. 

In sum, our findings emphasize the robust utility of SRQA in the implementation of highly accurate predictive algorithms for the detection of AF from ECG signals. In contrast to classical signal processing methods that have been applied to the analysis of ECG signals for predicting AF, SRQA is an ordinal approach focused on symbolic dynamics. This yields an insensitivity to extreme values and non-stationary processes, while also yielding a relatively small set of features that can be easily interpreted for descriptive, explanatory, or predictive purposes. Although this approach additionally minimizes preliminary analytical steps that might be needed in classical waveform analyses, e.g., subsequent spectral or power band analyses, SRQA as applied here nonetheless requires an initial pre-processing step of measuring RR intervals. This initial step is however easily automated, validated, and interpreted. Also, SRQA requires the selection of the free parameter m, named embedding dimension. The rule for choosing this parameter is given in Section 2.1.

## 5. Conclusions

The implementation of SRQA in this context will primarily be of interest in two broad applications: first, the symbolic dynamics measured in this procedure provide a novel tool to characterize dynamics in the cardiac system, and may also provide an effective tool for researchers to investigate biological factors related to AF. Accordingly, future studies should focus on the basic physiological implications of ECG signal dynamics identified via SRQA, such as associations with age, sex, and other biological parameters, as well as comorbid clinical conditions. Second, the computational efficiency, robust generalizability, and predictive accuracy of the algorithms characterized provide a promising framework that could easily be adapted to mobile software paired with a simple sensing device, as are commonly available in modern smartphones. In conjunction with ongoing medical care, mobile monitoring and detection of AF events could be used to complement clinical diagnoses and provide patients with more sensitive indicators of changing cardiac dynamics. Future studies should therefore also work to further develop the clinical utility of this method by extending this approach to a larger set of AF patients to provide a more complete validation of the model.

## Figures and Tables

**Table 1:** Threshold, specificity, sensitivity, and accuracy for window size 30, 60, 120 and 200.

	w = 30	w = 60	w = 120	w = 200
τ	0.414	0.448	0.513	0.510
**Se**	0.961	0.970	0.976	0.979
**Sp**	0.948	0.960	0.971	0.976
**ACC**	0.954	0.964	0.973	0.977

**w**, window’ size; τ threshold; **Se**, sensitivity; **Sp**, specificity; **ACC**, accuracy.

**Table 2:** Median and percentiles 25th and 75th values of threshold, Sensitivity, Specificity, and Accuracy for window size 30, 60, 120, and 200 of the 10-fold cross-validation.

	w = 30			w = 60			w = 120			w = 200		
	P_25_	Me	P_75_	P_25_	Me	P_75_	P_25_	Me	P_75_	P_25_	Me	P_75_
τ	0.399	0.417	0.420	0.419	0.457	0.465	0.460	0.479	0.510	0.498	0.505	0.526
**Se**	0.927	0.965	0.992	0.942	0.969	0.991	0.962	0.973	0.988	0.975	0.979	0.986
**Sp**	0.940	0.956	0.973	0.936	0.962	0.976	0.957	0.965	0.982	0.962	0.967	0.980
**ACC**	0.929	0.945	0.965	0.950	0.956	0.965	0.967	0.969	0.975	0.967	0.974	0.984

**w**, window’ size; τ threshold; **Se**, sensitivity; **Sp**, specificity; **ACC**, accuracy; **Me**, median; **P_25_** percentile 25th; **P_75_** percentile 75th.

## Appendix A

**Table A1:** Symbolic recurrence measures obtained from 50 RR interval data in normal sinus (N) and atrial fibrillation (AF) patients from Figure 3.

Symbolic Recurrence Measures	N	AF
SRR(0,1,2)	0.1567	0.0109
SRR(2,0,1)	0.0213	0.0213
SRR(1,2,0)	0.0109	0.0352
SRR(1,0,2)	0.0069	0.0278
SRR(0,2,1)	0.0017	0.0352
SRR(2,1,0)	0.0525	0.0434
D	0.6629	0.5341
ent(d)	0.8326	0.5873
$\mathrm{ent}\left(v_{\left(0,1,2\right)}\right)$	1.0397	0
$\mathrm{ent}\left(v_{\left(2,1,0\right)}\right)$	0.6365	0
${\bar{v}}_{\left(0,1,2\right)}$	4	2
${\bar{v}}_{\left(2,1,0\right)}$	2.333	2

**N**, normal sinus; **AF**, atrial fibrillation; SRR(π), symbolic recurrence rate for symbol π; **D**, percentage of recurrence points which form diagonal lines; ent(d), entropy of distribution of length of diagonal lines; $\mathrm{ent}\left(v_{\pi }\right)$, entropy of distribution of length of vertical lines; $v_{\pi }$, mean length of vertical lines of symbol π.

**Table A2:** Coefficients of the logistic model for the probability of AF for PhysioNet MIT-BIH Atrial Fibrillation database.

	w = 30	w = 60	w = 120	w = 200
	Coeff.	Coeff.	Coeff.	Coeff.
Intercept	9.25 **	16.97 **	27.04 **	36.44 **
$\bar{\mathrm{RR}}$	21.28 **	21.47 **	26.48 **	31.94 **
Me(RR)	−28.02 **	−28.77 **	−34.16 **	−39.54 **
CV	−32.14 **	−30.59 **	−28.02 **	−27.98 **
$V_{\mathrm{Me}}$	75.62 **	72.96 **	69.41 **	67.30 **
SRR(0,1,2)	−37.69 **	−63.26 **	−106.97 **	−133.84 **
SRR(2,0,1)	−27.90 **	−67.43 **	−119.86 **	−142.83 **
SRR(1,2,0)	−25.13 **	−58.88 **	−102.33 **	−128.14 **
SRR(1,0,2)	−27.53 **	−62.50 **	−108.35 **	−139.10 **
SRR(0,2,1)	−30.21 **	−68.41 **	−117.66 **	−145.21 **
SRR(2,1,0)	−27.42 **	−62.58 **	−114.04 **	−153.05 **
D	0.33	7.99 **	21.38 **	15.70 *
ent(d)	−2.91 **	−10.37 **	−21.45 **	−20.98 **
$\mathrm{ent}\left(v_{\left(0,1,2\right)}\right)$	−0.17	−0.51 **	−0.74 **	−0.64 *
$\mathrm{ent}\left(v_{\left(2,1,0\right)}\right)$	0.13	−0.31 *	−0.60 **	−0.68
${\bar{v}}_{\left(0,1,2\right)}$	−0.03	0.02	−0.19	−0.56
${\bar{v}}_{\left(2,1,0\right)}$	0.02	−0.04	−0.16	−0.20

w, window’ size; $\bar{\mathrm{RR}}$, mean RR interval; Me(RR), median RR interval; **CV**, coefficient of variation (standard deviation divided by the mean); $V_{\mathrm{Me}}$, coefficient of variation of the median; SRR(π), symbolic recurrence rate for symbol π; **D**, percentage of recurrence points which form diagonal lines; ent(d), entropy of distribution of length of diagonal lines; $\mathrm{ent}\left(v_{\pi }\right)$, enntropy of distribution of length of vertical lines; $v_{\pi }$, mean length of vertical lines of symbol π. Coefficient with a superimposed ∗ and ∗∗ are significant at 5% and 1% respectively, while those without asterisk mark are considered non-significant.
